# Microarray Analysis of Gene Expression Changes in Neuroplastin 65-Knockout Mice: Implications for Abnormal Cognition and Emotional Disorders

**DOI:** 10.1007/s12264-018-0251-5

**Published:** 2018-07-05

**Authors:** Huanhuan Li, Jiujiang Zeng, Liang Huang, Dandan Wu, Lifen Liu, Yutong Liu, Qionglan Yuan

**Affiliations:** 1 Department of Neurology, Shanghai Tongji Hospital, Tongji University School of Medicine, Shanghai, 200065 China; 2 Department of Radiology, University of Nebraska Medical Center, Omaha, NE 68198 USA

**Keywords:** Neuroplastin 65, Microarray analysis, Gene expression profile, Htr3a, Wnt

## Abstract

Neuroplastin 65 (Np65) is an immunoglobulin superfamily cell adhesion molecule involved in synaptic formation and plasticity. Our recent study showed that *Np65*-knockout (KO) mice exhibit abnormal cognition and emotional disorders. However, the underlying mechanisms remain unclear. In this study, we found 588 differentially-expressed genes in *Np65-*KO mice by microarray analysis. RT-PCR analysis also revealed the altered expression of genes associated with development and synaptic structure, such as *Cdh1*, *Htr3a*, and *Kcnj9*. In addition, the expression of Wnt-3, a Wnt protein involved in development, was decreased in *Np65*-KO mice as evidenced by western blotting. Surprisingly, MRI and DAPI staining showed a significant reduction in the lateral ventricular volume of *Np65*-KO mice. Together, these findings suggest that ablation of Np65 influences gene expression, which may contribute to abnormal brain development. These results provide clues to the mechanisms underlying the altered brain functions of Np65-deficient mice.

**Electronic supplementary material:**

The online version of this article (10.1007/s12264-018-0251-5) contains supplementary material, which is available to authorized users.

## Introduction

Neuroplastin (Np) is a member of the immunoglobulin (Ig) superfamily of cell adhesion molecules and exists in two isoforms, Np65 and Np55 [1]. Np65 contains extracellular Ig1-2-3 modules, while Np55 only contains extracellular Ig2-3. Thus, Np65 can be differentiated from Np55 by its extracellular Ig1. Np55 is expressed in various organs and cell types, whereas the expression of Np65 is brain-specific and restricted to neurons.

Np65 undergoes trans- and cis-homophilic bindings as well as several heterophilic bindings with fibroblast growth factor receptors, the α1 or α2 subunit of GABA_A_ receptors, and the basigin-monocarboxylate transporter [2–4]. Np65 has been implicated in the regulation of synaptic plasticity and the maintenance of excitatory/inhibitory balance. Antibodies specific for Np65 or recombinant Np65 block long-term potentiation (LTP) in the hippocampal CA1. The induction of LTP also increases the expression of Np65 in postsynaptic densities [5]. In addition, *Nptn*-deficient neurons exhibit impaired inhibitory transmission [6].

Previous studies have suggested that Np65 is associated with cognition and emotional states. Polymorphisms in the human *NPTN* gene have been shown to correlate with cortical thickness and intellectual abilities in adolescents as well as in patients with schizophrenia [7, 8]. *Nptn*-deficient mice exhibit retrograde amnesia, depressive-like behaviors, and decreased social interactions [9]. In addition, mutation of the *Nptn* gene results in deafness in mice, suggesting that *NPTN* is a novel deafness gene [10, 11]. We have previously demonstrated that *Np65* knock-out (KO) mice exhibit enhanced hippocampal-dependent spatial memory in the Morris water maze and step-through passive avoidance tests [12], but the underlying mechanisms were unclear. In this study, we used custom-designed microarray analysis to profile differentially-expressed genes in *Np65*-KO mice, in order to explain the altered brain functions in *Np65*-deficient mice.

## Materials and Methods

### Animals

The homozygous *Np65*-KO mice were obtained from engineered mouse models; this caused Np65-Ig1 deficiency in single chromosome as previously described [12]. Wild-type (WT) littermates served as controls. Animals were housed in a temperature-controlled environment under a 12 h light/dark cycle (08:00–20:00) with food and water *ad libitum*. All protocols complied with the National Institutes of Health Guide for the Care and Use of Laboratory Animals, and were approved by the Institutional Ethics Committee of Tongji University School of Medicine, and conformed to Directive 2010/63/EU and NIH guidelines.

### Microarray Experiments

Microarray analysis was performed as previously described [13]. Briefly, animals were sacrificed after deep anesthesia with intraperitoneal (i.p.) injection of 1% pentobarbital sodium (30 mg/kg). Hippocampi from adult *Np65*-KO mice (3 months old) and age-matched WT mice (*n* = 3/genotype) were dissected and immediately frozen in liquid nitrogen. The samples were stored at −80°C until use.

Total RNA was extracted from the hippocampal tissue using TRIzol (15596026, Thermo Fisher Scientific, Waltham, MA) and further purified with an RNeasy Mini Kit (74104, Qiagen, Hilden, Germany). RNA concentration and quality were evaluated by spectrophotometry (NanoDrop ND-1000, Thermo Fisher Scientific, Waltham, MA). One microgram of total RNA was amplified and labeled with a One-Color Quick Amp Labeling Kit (5190-0442, Agilent Technologies, Santa Clara, CA). The fluorescence-labeled cRNA was hybridized onto the Whole Mouse Genome Oligo Microarray (4 × 44K, Agilent Technologies, Santa Clara, CA) using the Agilent Gene Expression Hybridization Kit (5188-5242, Agilent Technologies, Santa Clara, CA). Chips were washed and scanned by a microarray scanner (G2565BA, Agilent Technologies, Santa Clara, CA). Raw data were then normalized and analyzed using the GeneSpring GX Software Package (v11.5, Agilent Technologies). The microarray experiment was performed with 3 biological and experimental repeats. Normalized values were used to screen for differentially-expressed genes from biological and experimental repeats before all replicates were combined. Genes with a fold-change of > 2.0 and a *P* value < 0.05 were selected for Gene Ontology (GO) and pathway analysis.

### Gene Ontology and Pathway Analysis

The fold-changes of differential expression were determined by the abundance ratio of *Np65*-KO and WT mice. Hierarchical clustering was used to analyze the differentially-expressed genes. GO analysis was applied to analyze the cellular components, biological functions, and biological processes of the differentially-expressed genes (www.geneontology.org). Pathway analysis was used to reveal significant Kyoto Encyclopedia of Genes and Genomes (KEGG) pathways of the differentially-expressed genes.

### Quantitative Real-Time Reverse-Transcription PCR

Quantitative real-time reverse-transcription PCR (RT-PCR) was performed as previously described [14]. Adult *Np65*-KO and WT mice (*n* = 4/genotype) were sacrificed after anesthesia with 1% pentobarbital sodium (30 mg/kg, i.p.) and the forebrain was harvested to extract total RNA using TRIzol (15596026, Thermo Fisher Scientific, Waltham, MA). RNA concentration and quality were determined by NanoDrop (ND-1000, Thermo Fisher Scientific, Waltham, MA). cDNA was generated using reverse transcriptase (PrimeScript™ RT reagent Kit, RR0747Q, Takara Bio, Tokyo, Japan). The first-strand cDNA was used as a template for RT-PCR analysis. The primers for RT-PCR analysis (Table 1) were designed by the NCBI primer designing tool [15] and synthesized by Sangon (Shanghai, China). Each RT-PCR reaction was carried out in a 20 μL volume using SYBR Green Master Mix (RR820Q, Takara Bio, Tokyo, Japan), started at 30 s at 95°C for initial denaturation, followed by 40 cycles of 5 s at 95°C and 34 s at 60°C in the ABI 7500 Real-Time PCR System. A total of 3 independent samples per subject were run in duplicate for RT-PCR. β-actin was used as the reference gene. The 2^*−* ΔΔCt^ method was used to determine the relative expression levels of genes.

**Table 1:** Primers used in RT-PCR.

Gene	NCBI Accession	Forward Primer	Reverse Primer
*Cdh1*	NM_009864	CAGCCGGTCTTTGAGGGATT	TGACGATGGTGTAGGCGATG
*Cdh4*	NM_009867	ACAACCGTCCCGAGTTCATC	TCATCTGCATCGTTGGCTGT
*Htr3a*	NM_013561	CAGACCACCTCCTGGCTAAC	GATGCTGTCTGTGGGGATGG
*Htr4*	NM_008313	ACGTCCTCATGCCCATTTCC	ACCACTGCAAGGAACGTGAG
*Kcnj9*	NM_008429	TCTTCTTCGTGCTCGCCTAC	CGAAGCCGTTGAGGTTGTTG
*Pla2g4e*	NM_177845	CTCCAACTGCCTACACCCAG	CCTCTGGGTTGAGTGGGAAC
*Xaf1*	NM_001037713	AGAGCCCATCCCAGAGTCAA	CAGATTGCTAAGCTGCACGG
*Lactb*	NM_030717	GGCTATGCAGACGTGGAGAA	CAGTTTAGCCAGAGCCACCA
*Actb*	NM_007393	GCTGTATTCCCCTCCATCGTG	AGTCCTTCTGACCCATTCCCA

### Western Blotting

Adult *Np65*-KO and WT mice (4 months old, *n* = 3/genotype) were used for western blotting. Briefly, animals were decapitated after deep anesthesia with 1% pentobarbital sodium (30 mg/kg i.p.). Forebrains were collected and frozen in nitrogen and then stored at −80°C until use. The total proteins were extracted using RIPA lysis buffer (P0013B, Beyotime) with 1 mmol/L PMSF (ST506, Beyotime). Protein concentrations were measured using the BCA Protein Assay Kit (P0010, Beyotime, Jiangsu, China), then 10 ng of total protein was separated by SDS-PAGE and transferred to the PVDF membrane. After blocking with 5% bovine serum albumin (BSA), the membranes were incubated overnight at 4°C with primary antibody against Wnt-3 (1:1,000, Santa Cruz Biotechnology, Dallas, TX) and mouse anti-glyceraldehyde-3-phosphate dehydrogenase (GAPDH, 1:1,000, Santa Cruz Biotechnology, Dallas, TX). Subsequently, the membranes were incubated with HRP-conjugated goat anti-mouse secondary antibody (1:1,000, Beyotime) for 2 h at room temperature. The labeled proteins were detected by using the ImageQuant LAS 4000mini system (GE Healthcare Life Sciences, Chicago, IL). The protein levels were normalized to that of GAPDH from three independent experiments.

### Magnetic Resonance Imaging (MRI)

A separate cohort of mice (4 months old, *n* = 5/genotype) was used in MRI analysis. MRI data were acquired with a 7.0 T animal MRI scanner (PharmaScan, Bruker Biospin GmbH, Germany) with 4-channel phased array coil. T2-weighed (T2-wt) MRI was performed using a rapid acquisition with relaxation enhancement (RARE) sequence with TR/TE = 4200/36 ms, RARE factor = 8, and averaging number = 3. The geometric parameters for the scan were: slice number = 18, slice thickness = 0.5 mm, matrix = 256 × 256, and FOV = 21 × 21 mm^2^.

### 4′,6-Diamidino-2-Phenylindole (DAPI) Staining

Adult *Np65*-KO and WT mice (*n* = 4, 2 months old) were anesthetized with 1% pentobarbital sodium intraperitoneally and perfused with 4% paraformaldehyde. The brain was removed, postfixed for 10 h–16 h, and cryoprotected in 20% sucrose. Coronal sections (10 μm, at the level of the lateral ventricle, 0 mm–2 mm from bregma) were prepared for DAPI staining. In brief, the sections were blocked in 5% BSA (B2064-100G, Sigma-Aldrich, St. Louis, MO) with 0.3% Triton X-100 (ST795, Beyotime), then incubated with DAPI (1:300, Beyotime) diluted in 1% BSA with 0.3% Triton X-100 for 10 min at room temperature. The sections were then rinsed with PBS and covered with Permount for fluorescent microscopy (Eclipse 80i, Nikon Corp., Tokyo, Japan).

### Statistics

Statistics were calculated using SPSS Statistics software (v22.0, IBM). All data are presented as mean ± SEM. Independent samples were tested by the unpaired Student’s *t-*test (two-tailed). The Mann-Whitney *U* test was used to determine the significance of data with an abnormal distribution or unequal variance. Statistical significance was set at *P* < 0.05.

## Results

### Microarray Analysis of Differentially-Expressed Genes in the Hippocampus of *Np65*-KO Mice

**Fig. 1:**
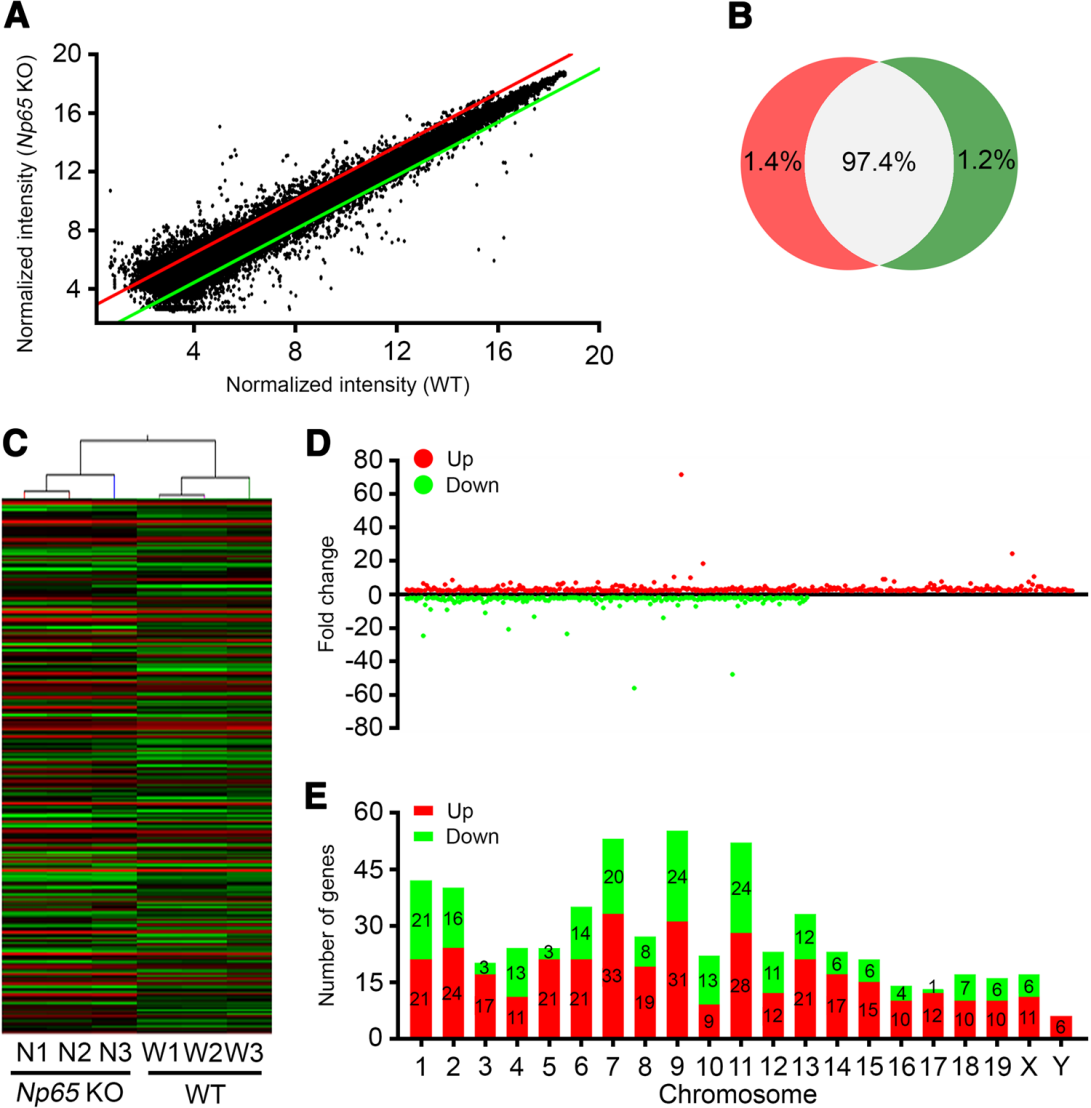
Differentially-expressed genes in the hippocampus of *Np65*-KO mice. **A** Scatter plot of normalized intensity derived from microarray chips with WT and *Np65*-KO mice. Dots above the red line denote upregulated genes, and dots below the green line denote downregulated genes. **B** Venn diagram showing the percentages of differentially-expressed genes categorized by fold-change. **C** Hierarchical clustering of differentially-expressed genes. N1–N3, *Np65*-KO mice; W1–W3, WT mice. **D** Fold changes of differentially-expressed genes in *Np65*-KO mice. **E** Chromosome distributions of differentially-expressed genes in *Np65*-KO mice. Red bars, numbers of upregulated genes; green bars, numbers of downregulated genes.

All genes are shown in a scatter plot with normalized intensity in Fig. 1A (details in Table S1). Of the 34397 targeted genes by the Mouse 4 × 44K Gene Chip, 481 (1.4%) were up-regulated and 418 (1.2%) were down-regulated by 2-fold in the *Np65*-KO mice (Fig. 1B). Using *P* < 0.05 as the criterion, 367 genes were significantly higher and 221 genes were significantly lower in *Np65*-KO mice as compared to age-matched WT mice (Fig. 1C and D, Table S2). These differentially-expressed genes were primarily located on chromosomes 7, 9 and 11. Notably, the *NPTN* gene resides on chromosome 9 (Fig. 1E).

### Gene Ontology and Pathway Analysis of Differentially-Expressed Genes

**Fig. 2:**
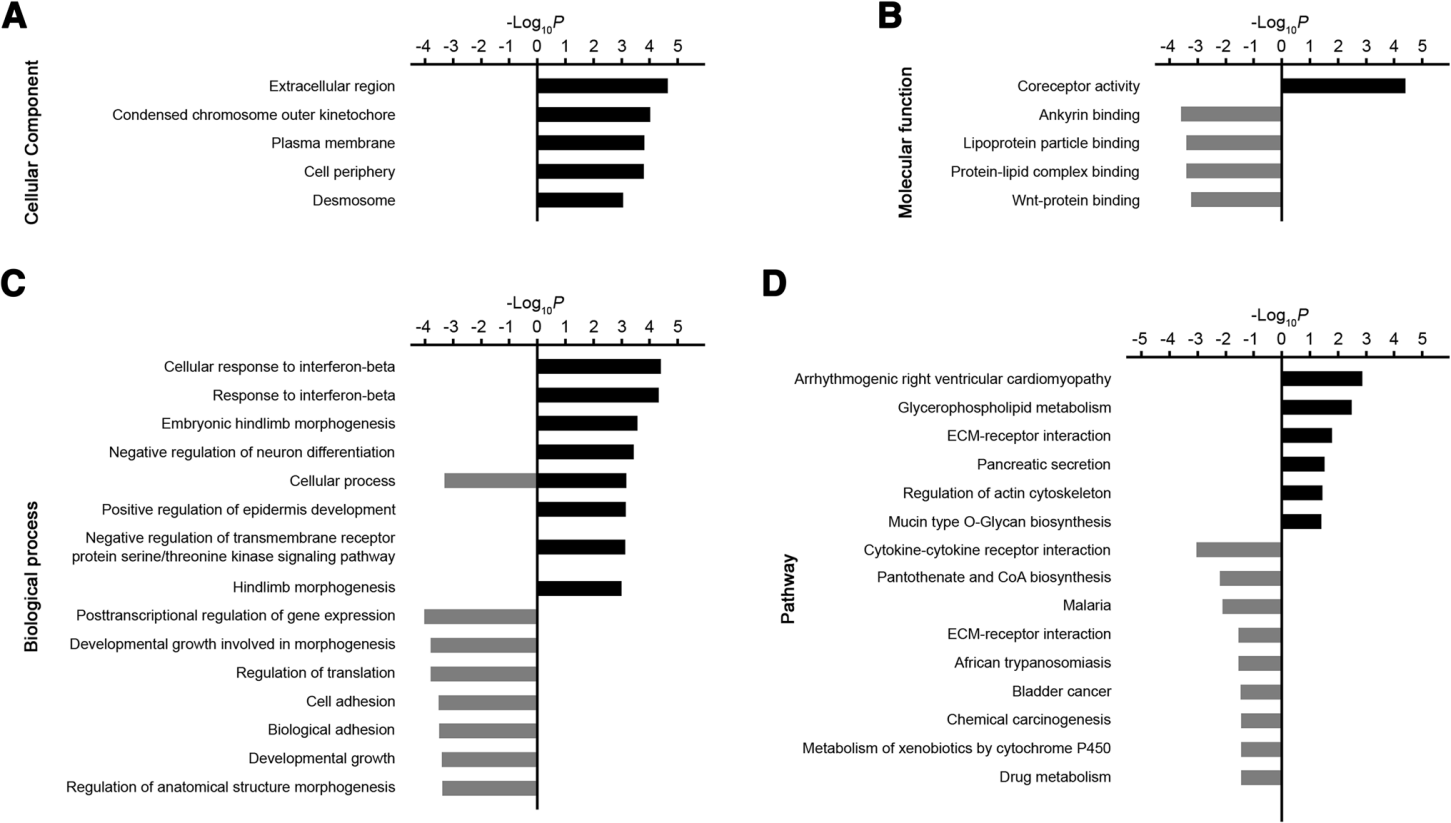
GO and pathway analysis of differentially-expressed genes in *Np65*-KO mice. **A**–**C** Cellular components (**A)**, molecular functions (**B)**, and biological processes (**C)** of differentially-expressed genes. **D** Significantly changed pathways in *Np65-*KO mice.

Using the criterion of *P* < 0.001, GO analysis showed that the upregulated genes were involved in several cellular components, including extracellular region, plasma membrane, and desmosome (Fig. 2A). The molecular functions of the upregulated genes were mainly associated with co-receptor activity. The down-regulated genes were involved in various binding actions, such as ankyrin binding, lipoprotein binding, and Wnt-protein binding (Fig. 2B). The main biological processes were the cellular response to interferon-beta, embryonic hindlimb morphogenesis, negative regulation of neuron differentiation, cellular process, positive regulation of epidermis development, and negative regulation of transmembrane receptor protein serine/threonine kinase signaling pathway. The downregulated genes were associated with several biological processes, including post-transcriptional regulation of gene expression, developmental growth involved in morphogenesis, regulation of translation, cell adhesion, developmental growth, and response to stimuli (Fig. 2C).

According to the pathway analysis, 8 pathways were significantly up-regulated in *Np65-*KO mice, including glycerophospholipid metabolism, pancreatic secretion, and regulation of actin cytoskeleton. Among the 9 down-regulated pathways, the most prominent was cytokine-cytokine receptor interaction. The other downregulated pathways were involved in bladder cancer, chemical carcinogenesis, and drug metabolism (Fig. 2D).

### Functional Analysis of Differentially-Expressed Genes in *Np65-*KO Mice

The differentially-expressed genes were divided into four categories: cell adhesion, development, neurotransmission and ion channel, and signal transduction. Previous studies have suggested that Np65 may interact with other cell adhesion molecules like fibroblast growth factor receptor (FGFR) to activate intracellular signaling [2]. Interestingly, the expression of several cell adhesion molecules was altered in *Np65-*KO mice. *Fgfr4*, immunoglobulin superfamily member 1 (*Igsf1*), interleukin 7 receptor (*Il7r*), and members of the integrin superfamily such as integrin alpha 1 (*Itga1*) and integrin alpha 9 (*Itga9*) were upregulated, while interleukin 1 receptor, type II (*Il1r2*) and protein tyrosine phosphatase receptor type D (*Ptprd*) were down-regulated in *Np65*-KO mice. In *Np65-*KO mice, the expression levels of genes associated with Ca^2+^ binding, including Ca^2+^-binding protein 5 (*Cabp5*), calbindin 1 (*Calb1*), and calmodulin-like 4 (*Calml4*) were significantly increased. However, several cadherins, including cadherin 1 (*Cdh1*), cadherin 4 (*Cdh4*), cadherin 6 (*Cdh6*), protocadherin 7 (*Pcdh7*), protocadherin 12 (*Pcdh12*), and protocadherin 17 (*Pchd17*) were down-regulated in *Np65-*KO mice (Table 2).

**Table 2:** Selected differentially-expressed genes in the hippocampus of *Np65*-KO mice.

Category	NCBI accession	Full name	Fold change	*P* value
Cell adhesion				
*Fgfr4*	NM_008011	Fibroblast growth factor receptor 4	2.0	0.004
*Igsf1*	NM_177591	Immunoglobulin superfamily, member 1	2.3	0.002
*Il7r*	NM_008372	Interleukin 7 receptor	2.1	0.006
*Il1r2*	NM_010555	Interleukin 1 receptor, type II	−2.1	0.002
*Ptprd*	XR_107615	Protein tyrosine phosphatase, receptor type D	−2.3	0.015
*Cdh1*	NM_009864	Cadherin 1	−2.4	0.010
*Cdh4*	NM_009867	Cadherin 4	−2.1	0.004
*Cdh6*	NM_007666	Cadherin 6	−2.2	0.043
*Pcdh7*	NM_001122758	Protocadherin 7	−5.8	0.020
*Pcdh12*	NM_017378	Protocadherin 12	−2.3	0.045
*Pcdh17*	NM_001013753	Protocadherin 17	−2.2	0.000
*Itga1*	NM_001033228	Integrin alpha 1	3.0	0.047
*Itga9*	NM_133721	Integrin alpha 9	10.4	0.000
Development				
*Ccr5*	NM_009917	Chemokine (C-C motif) receptor 5	−2.1	0.009
*Foxo3*	AK143198	Forkhead box O3	−2.0	0.004
*Mbp*	NM_010777	Myelin basic protein	−2.2	0.014
*Mef2c*	NM_025282	Myocyte enhancer factor 2C	−2.1	0.034
*Wif1*	NM_011915	Wnt inhibitory factor 1	−2.1	0.001
*Ntf3*	NM_001164034	Neurotrophin 3	3.2	0.044
*Gcm1*	NM_008103	Glial cells missing homolog 1	2.9	0.019
*Trp73*	NM_011642	Transformation related protein 73	2.3	0.040
*Aldh1a3*	NM_053080	Aldehyde dehydrogenase family 1, subfamily A3	−2.1	0.008
*Rpgrip1*	NM_023879	Retinitis pigmentosa GTPase regulator interacting protein 1	−2.5	0.020
*Crb1*	NM_133239	Crumbs homolog 1	−2.5	0.039
*Vsx1*	NM_054068	Visual system homeobox 1 homolog	−3.4	0.015
*Krt12*	NM_010661	Keratin 12	−2.9	0.000
*Sfrp5*	NM_018780	Secreted frizzled-related sequence protein 5	−2.0	0.006
*Myo7a*	NM_008663	Myosin VIIA	−2.8	0.040
*Cthrc1*	NM_026778	Collagen triple helix repeat containing 1	−2.1	0.000
Neurotransmission and ion channel				
*Cabp5*	NM_013877	Ca^2+^ binding protein 5	3.5	0.031
*Calb1*	AK038856	Calbindin 1	2.1	0.004
*Calml4*	NM_138304	Calmodulin-like 4	3.0	0.007
*Cplx2*	NM_009946	Complexin 2	2.1	0.033
*Htr4*	NM_008313	5-hydroxytryptamine (serotonin) receptor 4	2.4	0.043
*Htr3a*	NM_013561	5-hydroxytryptamine (serotonin) receptor 3A	−2.5	0.001
*Clca5*	NM_178697	Cl^–^ channel Ca^2+^ activated 5	2.9	0.000
*Kcnj9*	NM_008429	K^+^ inwardly-rectifying channel, subfamily J, member 9	4.2	0.000
Signal transduction				
*Map2k7*	NM_001042557	Mitogen-activated protein kinase kinase 7	−2.67	0.000
*Pla2g4e*	NM_177845	Phospholipase A2, group IVE	4.64	0.000
*Stk38l*	NM_172734	Serine/threonine kinase 38 like	4.80	0.013
*Ppp6r1*	NM_172894	Protein phosphatase 6, regulatory subunit 1	2.95	0.033
*Xaf1*	NM_001037713	XIAP associated factor 1	5.99	0.000
*Lactb*	NM_030717	Lactamase, beta	24.30	0.000

In addition, a subset of genes associated with neuronal development, such as chemokine (C-C motif) receptor 5 (*Ccr5*), forkhead box O3 (*Foxo3*), myelin basic protein (*Mbp*), myocyte enhance factor 2C (*Mef2c*), and Wnt inhibitory factor 1 (*Wif1*), were significantly downregulated in *Np65*-KO mice, while several genes involved in glial cell development, including neurotrophin 3 (*Ntf3*), glial cell missing homolog 1 (*Gcm1*), and transformation related protein 73 (*Trp73*), were upregulated. More interestingly, the expression of eye development-related genes was also altered in *Np65*-KO mice. The expression of aldehyde dehydrogenase family 1 subfamily A3 (*Aldh1a3*), retinitis pigmentosa GTPase regulator interacting protein 1 (*Rpgrip1*), crumbs homolog 1 (*Crb1*), visual system homeobox 1 homolog (*Vsx1*), keratin 12 (*Krt12*), and secreted frizzled-related sequence protein 5 (*Sfrp5*) were down-regulated. Myosin VIIA (*Myo7a*) and collagen triple helix repeat containing 1 (*Cthrc1*), which are associated with inner ear receptor cell development, were also decreased in *Np65*-KO mice (Table 2).

The expressions of genes related to the structure and function of synapses were also altered in *Np65-*KO mice. Notably, expression of the serotonin receptor 4 (*Htr4*) gene was significantly increased, whereas the expression of serotonin receptor 3A (*Htr3a*) was significantly decreased in *Np65*-KO mice. Moreover, two ion channel-related genes displayed significant downregulations in *Np65*-KO mice: Cl^–^ channel Ca^2+^ activated 5 (*Clca5*) and K^+^ inwardly-rectifying channel subfamily J member 9 (*Kcnj9*) (Table 2).

MAPK signaling is essential for various physiological and pathological processes, such as neural plasticity and memory. We found that the expression of mitogen-activated protein kinase kinase 7 (*Map2k7*) was significantly decreased, while phospholipase A2, group IVE (*Pla2g4e*), serine/threonine kinase 38 like (*Stk38l*), and protein phosphatase 6, regulatory subunit 1 (*Ppp6r1*) were significantly increased in *Np65*-KO mice. In addition, the expression level of X-linked inhibitor of apoptosis protein associated factor 1 (*Xaf1*), an apoptosis-promoting factor, was decreased. Most notably, β-lactamase (*Lactb*), which is involved in mitochondrial metabolism, was also significantly down-regulated in *Np65*-KO mice (24.30-fold, *P* < 0.001) (Table 2).

### RT-PCR Analysis of Differentially-Expressed Genes

**Fig. 3:**
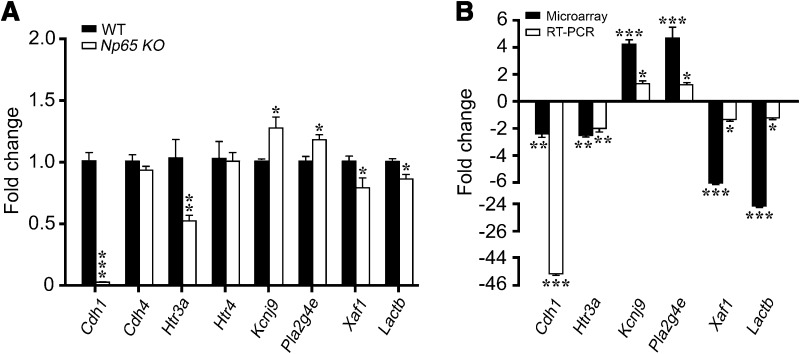
Relative expression levels of selected genes in *Np65*-KO and WT mice. **A** Results of quantitative real-time PCR (RT-PCR) (*n* = 4 mice). **B** RT-PCR and microarray experimental results for relative gene expression in *Np65*-KO and WT mice. The relative expression levels were calculated as the ratio of the target gene expression level to the β-actin expression level in the same sample. Fold changes are shown as mean ± SEM. *P < 0.05, **P < 0.01, ***P < 0.001.

Np65 is highly expressed in the hippocampus and other brain regions, such as cortex and striatum [1]. Therefore, 8 genes related to the functions of Np65 were further selected for RT-PCR analysis. The results showed that 6 of these were also significantly changed in the forebrain of *Np65*-KO mice (Fig. 3A), including the downregulated *Cdh1* (fold change, 45.34, *P* < 0.001), *Htr3a* (fold change, 1.94, *P* < 0.01), *Xaf1* and *Lactb*, and the increased *Kcnj9* (fold change, 1.26, *P* < 0.05) and *Pla2g4e* (fold change, 1.17, *P* < 0.05) (Fig. 3B).

### Decreased Expression of Wnt-3 in *Np65*-KO Mice

**Fig. 4:**
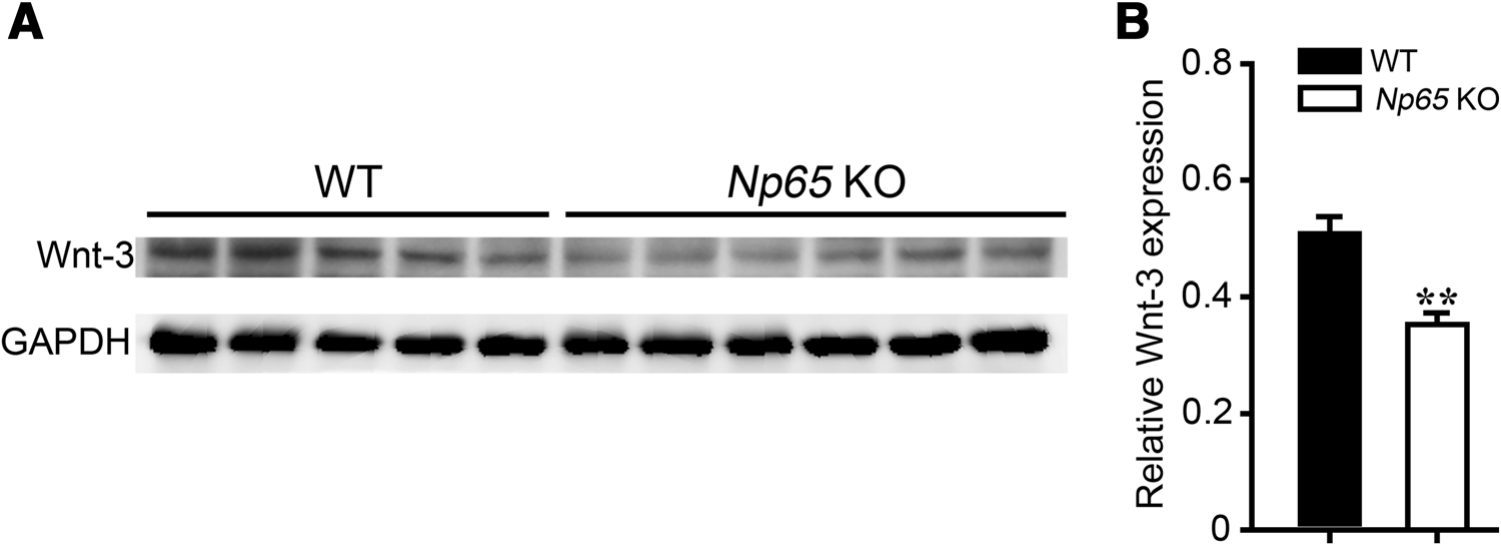
Reduced expression of Wnt-3 in *Np65*-KO mice. **A** Representative Wnt-3 bands from the forebrain of WT (*n* = 5) and *Np65*-KO mice (*n* = 6). **B** Quantitative results of western blotting analysis showed that the expression level of Wnt-3 was significantly decreased in the forebrain of *Np65*-KO mice. All data are presented as mean ± SEM. **P < 0.01.

Microarray and RT-PCR analysis showed that the expression levels of several genes associated with development were altered in *Np65-*KO mice. Some differentially-expressed genes, such as *Wif1* and *Cdh1*, are involved in Wnt signaling. Wnt signaling is a crucial regulator of many developmental processes, such as cell proliferation, maintenance of stem cells, and cell fate determination [16]. Therefore, we examined the protein level of Wnt-3 in *Np65*-KO mice by western blotting. The results showed that the protein level of Wnt-3 was significantly lower in the forebrain of *Np65*-KO mice than in WT mice (Fig. 4).

### Reduced Lateral Ventricles in *Np65*-KO Mice

**Fig. 5:**
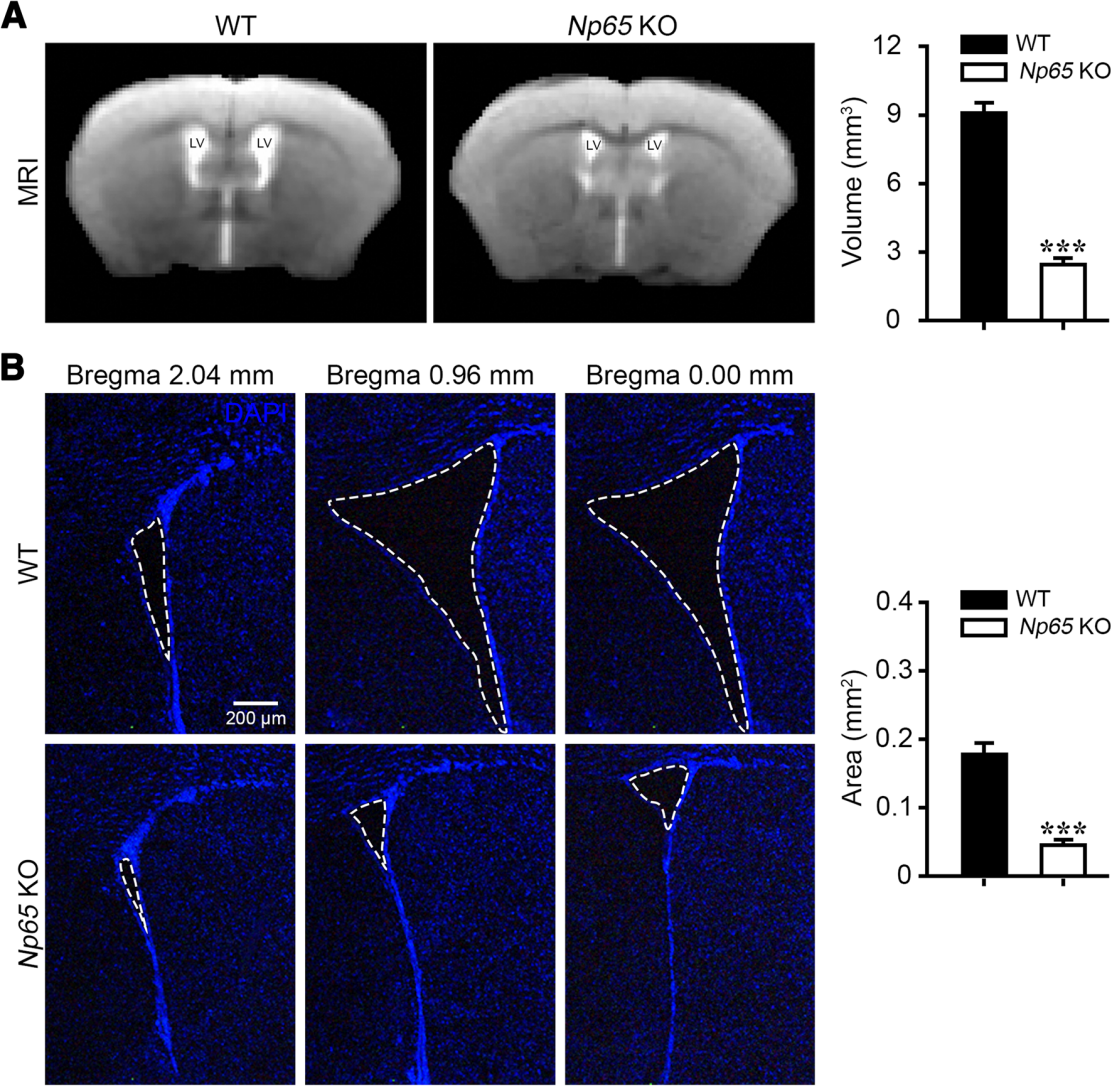
Reduction in lateral ventricles in *Np65*-KO mice. **A** T2-wt MRI showing a significant reduction in the lateral ventricles (LV) compared to WT mice. **B** DAPI staining showing a significant reduction in the lateral ventricles in coronal sections from adult *Np65*-KO mice compared to WT mice. Scale bar, 200 µm. ***P < 0.001.

Given that the dysfunction of Wnt signaling may influence brain development, we then assessed whether ablation of Np65 affects the brain morphology of mice. T2-wt images were used to assess region-specific volume changes. The gross brain architecture was not affected in *Np65*-KO mice. MRI morphometry also showed normal anatomy of the cerebral cortex, hippocampus, thalamus, hypothalamus, basal ganglia, and caudatoputamen of *Np65-*KO mice. However, the lateral ventricular volume was significantly reduced compared to WT mice (Fig. 5A), and this was further confirmed by DAPI staining (Fig. 5B). Thus, these results suggested that the absence of Np65 leads to altered architecture of the mouse brain.

## Discussion

Np65 is specifically expressed in the brain and has been reported to mediate several cellular processes including cell-cell adhesion, neurite outgrowth, and synaptic plasticity [2, 5, 17, 18]. Our previous studies have shown that *Np65*-KO mice exhibit abnormal cognitive and emotional behaviors [12]. To investigate the underlying mechanisms, we further analyzed the gene expression profiles in *Np65*-KO mice in this study. Our microarray analysis demonstrated a large number of differentially-expressed genes in *Np65*-KO mice; these genes are crucially involved in development, ion channels, neurotransmission, and signal transduction.

Our study identified many differentially-expressed genes involved in neuronal development, such as the decreased expressions of *Cdh1*, *Ccr5*, *Foxo3*, *Mbp*, *Wif1*, and *Mef2c*, as well as upregulation of *Ntf3*, *Gcm1*, and *Trp73*, implying that Np65 deletion affects the configuration of the brain. Coincidently, T2-wt MRI morphometry and brain slices stained with DAPI showed a significant reduction in lateral ventricular volume in *Np65*-KO mice compared to WT mice. The expression of Wnt-3 was significantly decreased in *Np65*-KO mice. Wnt signaling is a crucial regulator of developmental processes like cell proliferation and cell fate determination [16]. Dysregulation of Wnt signaling may contribute to neuropsychiatric disorders, such as depression and schizophrenia [19]. In this study, our findings suggested that Np65 deletion affects the Wnt signaling pathway by decreasing Wnt expression. Together, these differentially-expressed genes associated with development may contribute, at least in part, to changes in the ventricles and abnormal behaviors in *Np65*-KO mice.

Recent studies have reported that mutation of the *NPTN* gene results in deafness in mice [10]. It has been reported that Np65 may regulate the properties of synapses connecting the inner hair cells with spiral ganglion neurons [10]. Intriguingly, Zeng *et al.* reported that Np55 is expressed in stereocilia of outer but not inner hair cells and affects interactions of stereocilia with the tectorial membrane and cochlear amplification in mice with *NPTN* mutation [11]. Together, these recent findings clearly confirm *NPTN* as a novel deafness gene. Consistent with their reports, our microarray analysis showed that the genes associated with inner ear receptor cell development, myosin VIIA (*Myo7a*) and collagen triple helix repeat containing 1 (*Cthrc1*) were significantly decreased in *Np65*-KO mice, supporting the hypothesis that Np65 is involved in hearing.

It has been shown that Np65 is linked with ribbon synapse formation in the plexiform layers of the rat retina [20]. Retinal function, as assessed using the electroretinogram, is unaffected by the absence of *NPTN* [10]. Surprisingly, the involvement of Np65 in vision was demonstrated using the pupillary light reflex and flash visual evoked potentials (our unpublished data). In agreement with our finding, our microarray analysis showed that the expression of eye development-related genes, including *Aldh1a3*, *Rpgrip1*, *Crb1*, *Vsx1*, *Krt12*, and *Sfrp5*, was significantly downregulated in *Np65-*KO mice. Although these alterations in eye-development genes need to be confirmed, the reduced amplitude of the pupil in the pupillary light reflex and reduced first negative and positive amplitude of flash visual evoked potentials (our unpublished data) suggest that Np65 plays roles in vision.

Our previous studies have shown that *Np65*-KO mice appear to show enhanced memory in the Morris water maze and increased anxiety [12]. Central 5-hydroxytryptamine (5-HT) activity is involved in emotional and cognitive activities [21, 22]. Generally, stimulation of central 5-HT activity impairs cognition, while its inhibition enhances cognition in rodent models. Tropisetron, a selective 5-HT_3_ receptor antagonist, has been confirmed to reverse the cognitive deficit in rats injected with Aβ (1–42) [23]. In addition, central 5-HT activity is closely associated with anxiety [24–27]. Among the 5-HT receptors, 5-HT_3_ is the only ligand-gated ion channel that increases intracellular cations such as Ca^2+^, Na^+^, and K^+^. Stimulation of 5-HT_3_ receptors induces the rapid and transient depolarization of neurons. 5-HT receptor 3A-null mice exhibit anxiolytic behaviors, indicating that this receptor influences anxiety-like behavior [28]. More surprisingly, microarray and RT-PCR analysis demonstrated that *Htr3a* mRNA was significantly reduced in *Np65*-KO mice. How deletion of Np65 affects the expression of *Htr3a* remains to be determined. To date, the decreased expression of *Htr3a* may explain, at least in part, the changed cognitive and anxiety behaviors in *Np65*-KO mice.

In conclusion, the present study demonstrates that a large number of genes are differentially expressed in *Np65*-KO mice. Notably, microarray analysis in *Np65*-KO mice revealed altered expression of *Htr3a* and genes associated with development, hearing, and vision, which may provide important insights for understanding the role of Np65 in brain development as well as brain functions like cognition and emotion.

## Electronic supplementary material

Below is the link to the electronic supplementary material.

Supplementary material 1 (XLS 428 kb)

Supplementary material 2 (XLSX 11345 kb)
